# Curcumin-Loaded Lipid Nanocarriers: A Targeted Approach for Combating Oxidative Stress in Skin Applications

**DOI:** 10.3390/pharmaceutics17020144

**Published:** 2025-01-21

**Authors:** Aggeliki Liakopoulou, Sophia Letsiou, Konstantinos Avgoustakis, George P. Patrinos, Fotini N. Lamari, Sophia Hatziantoniou

**Affiliations:** 1 Laboratory of Pharmaceutical Technology, Department of Pharmacy, School of Health Sciences, University of Patras, GR-265 04 Patras, Greece; aggelikiliakopoulou27@gmail.com (A.L.); avgoust@upatras.gr (K.A.); 2 Laboratory of Pharmacogenomics and Individualized Therapy, Department of Pharmacy, School of Health Sciences, University of Patras, GR-265 04 Patras, Greece; sletsiou@gmail.com (S.L.); gpatrinos@upatras.gr (G.P.P.); 3 Laboratory of Pharmacognosy and Chemistry of Natural Products, Department of Pharmacy, School of Health Sciences, University of Patras, GR-265 04 Patras, Greece; flam@upatras.gr

**Keywords:** lipid nanoparticles, solid lipid nanoparticles, nanostructured lipid carriers, nanoemulsions, curcumin, antioxidant activity, skin penetration

## Abstract

**Background/Objectives:** Oxidative stress significantly impacts skin health, contributing to conditions like aging, pigmentation, and inflammatory disorders. Curcumin, with its potent antioxidant properties, faces challenges of low solubility, stability, and bioavailability. This study aimed to encapsulate curcumin in three lipid nanocarriers—solid lipid nanoparticles (SLNs), nanostructured lipid carriers (NLCs), and nanoemulsions (NEs)—to enhance its stability, bioavailability, and antioxidant efficacy for potential therapeutic applications in oxidative-stress-related skin disorders. **Methods:** The lipid nanocarriers were characterized for size, polydispersity index, ζ-potential, and encapsulation efficiency. Stability tests under various conditions and antioxidant activity assays (DPPH and FRAP methods) were conducted. Cytotoxicity in human dermal fibroblasts was assessed using MTT assays, while the expression of key antioxidant genes was evaluated in human dermal fibroblasts under oxidative stress. Skin penetration studies were performed to analyze curcumin’s distribution across the stratum corneum layers. **Results:** All nanocarriers demonstrated high encapsulation efficiency and stability over 90 days. NLCs exhibited superior long-term stability and enhanced skin penetration, while NE formulations facilitated rapid antioxidant effects. Antioxidant assays confirmed that curcumin encapsulation preserved and enhanced its bioactivity, particularly in NLCs. Gene expression analysis revealed upregulation of key antioxidant markers (*GPX1*, *GPX4*, *SOD1*, *KEAP1*, and *NRF2*) with curcumin-loaded nanocarriers under oxidative and non-oxidative conditions. Cytotoxicity studies confirmed biocompatibility across all formulations. **Conclusions:** Lipid nanocarriers effectively enhance curcumin’s stability, antioxidant activity, and skin penetration, presenting a targeted strategy for managing oxidative stress in skin applications. Their versatility offers opportunities for tailored therapeutic formulations addressing specific skin conditions, from chronic disorders like psoriasis to acute stress responses such as sunburn.

## 1. Introduction

Oxidative stress, characterized by an imbalance between reactive oxygen species (ROS) production and antioxidant defenses, has been recognized as a primary contributor to skin aging and various skin disorders, including psoriasis, acne, and seborrheic dermatitis. This imbalance results in excessive ROS accumulation, leading to cellular damage, apoptosis, and the degradation of structural proteins such as collagen and elastin, which are essential for maintaining skin integrity and resilience [1,2]. Furthermore, prolonged exposure to environmental stressors like ultraviolet (UV) radiation intensifies oxidative stress, accelerating skin aging and increasing susceptibility to inflammation and other skin pathologies. Understanding how to mitigate ROS-induced damage is vital for protecting and restoring skin health.

Natural antioxidants derived from plant-based sources have shown promise for treating oxidative-stress-related skin conditions [3]. These include polyphenols, essential oils, and vitamins, which are widely used in cosmetics, pharmaceuticals, and functional foods due to their safety and broad therapeutic properties [4].

Among these, curcumin (CUR), a diaryleptanoid derived from *Curcuma longa* L., has attracted attention due to its diverse pharmacological activities, including antioxidant, anti-inflammatory, antimicrobial, and wound-healing properties [5,6,7,8]. Recent studies have demonstrated CUR’s potential in managing skin conditions such as psoriasis, atopic dermatitis, UVB-induced skin damage, and its ability to enhance collagen deposition and accelerate wound healing [9]. Notably CUR has shown significant efficacy in inhibiting ROS formation and lipid peroxidation, particularly when combined with other antioxidants like resveratrol and gallic acid [10].

Despite its therapeutic potential, CUR’s clinical application is hindered by its physicochemical limitations, including poor water solubility, hydrophobicity, photosensitivity, and instability in alkaline environments [6]. While topical administration of CUR offers advantages over oral delivery, such as localized effects and bypassing first-pass metabolism, limited skin penetration poses a significant challenge. Encapsulation of CUR into lipid nanocarriers has emerged as a promising strategy to overcome these limitations, improving CUR’s solubility, stability, and skin penetration while enabling controlled release and reducing toxicity [6,11,12,13,14].

Lipid nanocarriers, including solid lipid nanoparticles (SLNs), nanostructured lipid carriers (NLCs), and nanoemulsions (NEs), offer distinct advantages for CUR [15,16]. SLNs, composed of solid lipids, provide a stable environment for encapsulation and sustained release. However, during storage, these lipids may undergo recrystallization, which can lead to drug expulsion and particle aggregation due to deformation. Despite these challenges, SLNs are valued for their high physical stability and sustained release properties, making them advantageous for prolonged therapeutic effects [16]. NLCs, which combine solid and liquid lipids, exhibit enhanced flexibility, reduced crystallinity, and improved stability compared to SLNs. This unique composition not only enhances drug loading efficiency but also reduces the risk of recrystallization and particle aggregation, thereby improving nanoparticle stability and potentially facilitating skin penetration [17]. NEs, fine dispersions of oil in water stabilized by surfactants, are known for their ease of production and ability to enhance bioavailability [18]. Despite the known stability challenges of NEs, such as Ostwald ripening and phase separation, these issues may be minimized through careful formulation strategies, including the selection of suitable surfactants and co-surfactants, optimization of the oil-to-water ratio, and the use of high-energy emulsification techniques.

This study aims to evaluate the potential of SLNs, NLCs, and NEs as delivery systems for CUR in the context of oxidative-stress-related skin conditions. By encapsulating curcumin within these nanocarriers, we investigated how differences in lipid matrix fluidity affect their physicochemical properties, stability, and antioxidant activity. Additionally, to simulate oxidative stress conditions, we exposed a 2D cell-based model of human dermal fibroblasts to oxidative stress under hydrogen peroxide (H_2_O_2_) and assessed the effects of each curcumin-loaded nanocarrier on cell viability and gene expression of key antioxidant markers involved in skin processes such as hydration, aging, and pigmentation. These findings provide valuable insights into the design of lipid nanocarriers for the topical treatment of skin disorders associated with oxidative stress, including psoriasis, acne, and seborrheic dermatitis.

## 2. Materials and Methods

Curcumin (purity ≥ 98%) (Acros Organics, Geel, Belgium), ethanol (purity ≥ 99.8%) (Acros Organics, NJ, USA), methanol (purity ≥ 99.8%) (Honeywell Riedel-de Haën, Seelze, Germany), phosphate-buffered saline (PBS) (Sigma Aldrich, Darmstadt, Germany), L-ascorbic acid (Sigma Aldrich, Darmstadt, Germany), and citric acid (Chemco, Pharmaceutical Industry, Ilion, Attica, Greece) were of analytical grade; saboderm TCC (SABO S.p. A., Bergamo, Italy, Caprylic/Capric Triglycerides), softisan 100 (Sasol GmbH, Hamburg, Germany, INCI: Hydrogenated Coco-Glycerides), solutol HS 15 (BASF, Ludwigshafen, Germany, INCI: 111 Macrogol (15)-hydroxystearate), and Emulmetik™ 900 (Lucas Meyer Cosmetics, Champlan, France, INCI: Lecithin) were of cosmetic grade, and water for injection (WFI) (Demo S.A., Pharmaceutical Industry, Kryoneri, Attica, Greece) was of pharmaceutical grade.

### 2.1. Preparation of Lipid Nanoparticles and Nanoemulsions

Three types of nanocarriers loaded with curcumin (CUR) were prepared and designated as SLN.CUR, NLC.CUR, and NE.CUR. Additionally, a corresponding set of empty nanocarriers (SLNs, NLCs, NEs) were prepared for comparison. Each carrier differed in the viscosity of its internal phase, regulated by the physical state of the triglyceride contained. Nanocarrier preparation was conducted using the hot emulsification method followed by sonication, as previously described [19]. The lipid-phase concentration in all formulations was 3% *w*/*w*. The compositions of the lipid phase for each nanocarrier were as follows:

SLNs (solid lipid nanoparticles): softisan 100/Emulmetik™ 900 in a 1:1 *w*/*w* ratio.

NLCs (nanostructured lipid carriers): saboderm TCC/softisan 100/Emulmetik™ 900 in a 1:2:3 *w*/*w* ratio.

NEs (nanoemulsions): saboderm TCC/Emulmetik™ 900 in a 1:1 *w*/*w* ratio.

Initially, a conventional emulsion was prepared by heating the aqueous and lipid phases separately (65–70 °C) and adding the aqueous phase dropwise to the lipid phase with continuous stirring (200–300 rpm) on a thermal/magnetic stirrer (ARE 5, Velp Scientifica, Usmate Velate (MB), Italy). For the CUR-loaded nanocarriers, the precise quantity of CUR was weighed to achieve a final concentration of 0.05% (*w*/*w*) and added to the melted lipid phase before emulsification. The emulsion was then cooled under stirring (500 rpm, 30 min) until it reached room temperature. Subsequently, the prepared emulsion was further homogenized by sonication (Vibra-Cell VCX 130PB, Sonics & Materials, Inc., Newtown, CT, USA) with 83% amplitude for 1 min/mL until the nanocarriers were formed. This was followed by cooling using a vortex mixer (8000 rpm, Vortex-Genie 2, Scientific Industries, Bohemia, New York, NY, USA) to room temperature (RT) and storage at 4 °C in light-protected vials.

### 2.2. Physicochemical Characterization of Nanoparticles

#### 2.2.1. Particle Size and ζ-Potential Measurement

Dynamic light scattering (DLS) was used to calculate the average particle size and polydispersity index (PdI) of the nanocarriers. The measuring device (Zetasizer Nano-ZS, Malvern Panalytical Ltd., Malvern, UK) was equipped with a He-Ne laser beam (633 nm) at a scattering angle of 173°. Polystyrene latex particles were used for instrument calibration, the refractive index (RI) was set to that of water (1.333), and measurements were conducted at 25 °C. The size values resulted from three independent measurements, each averaging 12 autocorrelation diagrams and fitting procedures. Electrophoretic light scattering (ELS) was used to determine the ζ-potential of dispersed particles in the prepared samples with the same measuring instrument.

#### 2.2.2. Morphological Study of Nanoparticles by Transmission Electron Microscopy

The morphology of the CUR-loaded and empty nanocarriers was examined using a transmission electron microscope (TEM) (JEM-2100F, JEOL, Tokyo, Japan). A drop of each dispersion was placed on a carbon-coated copper grid, and the excess was removed with filter paper after 5 min. A drop of 2% phosphotungstic acid hydrate (PTA) (Sigma-Aldrich, Steinheim, Germany) solution was added for enhanced contrast via negative staining of the nanoparticles, and the samples were allowed to dry at room temperature (approximately 5 min). TEM images were taken at an accelerating voltage of 200 kV [20]. Particle size was measured using the original ImageJ [21] software.

#### 2.2.3. Determination of CUR Loading and Encapsulation Efficiency in Nanoparticles

The CUR content of the samples was determined by spectrophotometry (UV-1800 UV–Vis Spectrophotometer, SHIMADZU, Kyoto, Japan). After diluting each sample with methanol, the absorbance at 425 nm was measured, and the corresponding concentrations were calculated constructing a calibration curve using standard CUR solutions in MeOH.

Encapsulation efficiency (EE%) and loading of CUR in the three nanocarriers was achieved after the removal of non-incorporated molecules via size exclusion chromatography, with Sephadex G-75 as the stationary phase and WFI as the mobile phase.

This allowed for the calculation of the CUR content in each sample (before column) and the measurement after the column (mass of loaded CUR). From these values and the theoretical CUR content (based on initial weighting), the encapsulation efficiency (% EE), actual loading (% loading capacity, % LC), and theoretical loading capacity (% TL) of CUR in the nanoparticles were calculated according to Equations (1), (2), and (3), respectively.

% EE = (mass of loaded CUR)/(initial mass of CUR) × 100,(1)

%LC = (mass of loaded CUR)/(mass CUR loaded nanoparticles) × 100,(2)

% TL = (initial mass of CUR)/(initial mass of carrier + initial mass of CUR) × 100,(3)

where mass of loaded CUR: the mass of CUR determined after passing the samples through a molecular exclusion column; initial mass of CUR: the mass of CUR used for the preparation of nanocarriers; and mass of carrier: the lipid mixture mass.

### 2.3. Stability Studies of Nanoparticles

#### 2.3.1. Centrifugation Test

Immediately after preparation, all nanocarriers were subjected to centrifugation (Centrifuge Hermle Z32HK, Hermle Labortechnik GmbH, Wehingen, Germany). A small quantity (1–1.5 mL) from each sample was placed in Eppendorf tubes and centrifuged at 25 °C at a speed of 5000 rpm for 15 min. At the end of the test, all samples were visually inspected for possible phase separation.

#### 2.3.2. Accelerated Aging Test

All samples, one day after preparation, were subjected to an accelerated aging test. This test included three heating cycles at 45 °C and cooling cycles at 25 °C. Parameters measured for evaluating sample stability included the particle size, PDI, ζ-potential, and CUR content on the 1st and 7th days (final day) of testing.

#### 2.3.3. Storage at Constant Temperature (4 °C)

The colloidal stability of the dispersions was assessed after storage at a constant temperature of 4 °C in light-protected containers by measuring the particle size, PDI, ζ-potential, and CUR content at predetermined intervals (1, 8, 15, 30, 60, 90 days) for a period of 90 days.

### 2.4. Evaluation of Antioxidant Activity of Nanoparticles

#### 2.4.1. Antioxidant Activity via DPPH Radical Scavenging Mechanism

The protocol for evaluating antioxidant activity followed the methodology previously described, with minor modifications [22,23]. This method used the stable free radical 2,2-diphenyl-1-picrylhydrazyl (DPPH), which has a characteristic deep violet color (due to its delocalized electron), and upon reacting with other radicals, electrons, or hydrogen atoms, it loses color, thus reducing absorption in the solution (540 nm).

This method investigated the impact of CUR encapsulation in the nanocarriers on the biomolecule’s antioxidant activity. A 0.20 mM DPPH solution in MeOH was prepared. Aqueous and ascorbic acid solutions in methanol were used as controls for the dispersions and free curcumin, respectively. All reactions were conducted on 96-well plates. Reaction solutions contained 20 μL of sample or ascorbic acid solution and 80 μL of DPPH solution. Control samples contained 20 μL of sample or ascorbic acid solution and 80 μL of MeOH. Blank samples contained 20 μL of water and 80 μL of MeOH. For free CUR, the blank contained 100 μL MeOH.

To calculate the radical scavenging activity (% RSA) of CUR encapsulated in the nanocarriers and free CUR, standard curves of ascorbic acid in water and methanol were constructed based on sample solubility. The initial concentration of CUR in all samples compared was 50.0 μg/mL.

Due to the photosensitivity of both CUR and free radicals, the plate was covered with aluminum foil and incubated in darkness at room temperature for 30 min. After 30 min, sample absorbance at 540 nm was measured using a Sunrise^®^ microplate reader (Tecan Trading AG, Männedorf, Switzerland). The percentage of radical inhibition was calculated using Equation (4):

RSA (%) = [1 − (Abs(sample)/Abs(control)] × 100,(4)

#### 2.4.2. Antioxidant Activity via Ferric-Cation-Reduction Mechanism (FRAP Method)

The method developed by Benzie and Strain (1999) measures reducing power in plasma and was adapted with minor modifications [24]. It is based on the principle of reducing the Fe^+3^-2,4,6-tri(2-pyridyl)-1,3,5-triazine (Fe^+3^-TPTZ) complex to its divalent form (Fe^+2^-TPTZ) by sample antioxidants in acidic pH, resulting in a blue color with a maximum absorbance at 595 nm. Absorbance changes are linked to the antioxidant capacity of the sample [25,26]. Reactions were performed on 96-well plates.

The test reagent was prepared by mixing, in a 10:1:1 ratio, the following solutions: (a) 0.3 M acetate buffer pH = 3.6 (0.31 g CH_3_COONa and 1.5 mL CH_3_COOH per 100 mL aqueous solution), (b) 10 mM TPTZ solution in 40 mM hydrochloric acid, and (c) 20 mM ferric chloride hexahydrate solution. Ascorbic acid solutions, either aqueous or methanolic, were used as standards for the dispersions and free curcumin, respectively. Reaction solutions contained 10 μL of sample or ascorbic acid solution and 190 μL of FRAP reagent. Blank samples contained 10 μL of sample or ascorbic acid solution and 190 μL of water.

Due to CUR’s photosensitivity, the plate was carefully covered with aluminum foil and then incubated in a water bath at 37 °C for exactly 5 min. Immediately afterwards, absorbance at 595 nm was measured using a Sunrise^®^ microplate reader (Tecan Trading AG, Männedorf, Switzerland). The results are expressed as ascorbic acid equivalents (μg/mL) using standard curves.

### 2.5. Cell Culture, Cytotoxicity, and Molecular Analysis Protocols

#### 2.5.1. Reagents and Cell Culture

Primary Normal Human Dermal Fibroblasts (NHDFs) (Lonza, Walkersville, MD, USA) isolated from normal human adult skin were used. Cells were cultured following the producer’s recommendations and grown in FGM-2 BulletKit media containing 2% serum and subcultured until they reached 70–80% confluence.

#### 2.5.2. Cytotoxicity Assessments

Cell viability was determined using an MTT colorimetric assay kit (Vybrant^®^ MTT Cell Proliferation Assay Kit, Thermo Fisher Scientific, Waltham, MA, USA) following the manufacturer’s protocol. Briefly, after cell treatment (with or without oxidative stress conditions), a mix of 100 μL of FBM and 10 μL of MTT labeling reagent (5 mg/mL) was added in each well, and the plate was incubated for 3 h at 37 °C. Subsequently, 50 μL of DMSO was added, and the plate was incubated for 10 min at 37 °C. Finally, the absorbance of the reaction solution was measured at 570 nm using a microplate reader (infinite 200M Pro, Tecan, Männedorf, Switzerland). Measurements were performed, and % cell viability was determined using Equation (5):

Cell viability (%) = Mean OD/Control OD × 100%,(5)

Different concentrations (0.5–100 μg/mL) were assessed for cell viability for all the samples.

#### 2.5.3. RNA Isolation, cDNA Synthesis, and PCR Analysis

For total RNA isolation, a Nucleospin RNA kit (Macherey-Nagel, Düren, Germany) was used according to the manufacturer’s instructions. Total RNA (500 ng) was used for synthesis of complementary DNA (cDNA) using a SuperScript™ First-Strand Synthesis kit (Invitrogen, Carlsbad, CA, USA) in a total reaction volume of 20 μL by following the manufacturer’s instructions.

RT-qPCR was used to assess changes at a molecular level based on the method described before [14]. Briefly, quantitative RT-PCR reactions were performed using Kapa Sybr Fast qPCR Master Mix (Kapa Biosystems, Inc., Wilmington, MA, USA), and the RT-qPCR cycle was performed in a Light Cycler^®^ 96 system (Roche Diagnostics, Santa Clara, CA, USA). The primer pairs were used at a final concentration of 0.5 μΜ in a 1 μL of the cDNA template. For the relative gene expression, the comparative threshold cycle (Ct) method was used, and normalization was based on two reference genes: Actin beta (*ACTB*) and Glyceraldehyde-3-phosphate dehydrogenase (*GAPDH*) (Table S1).

### 2.6. Assessment of the Nanocarriers’ Penetration of the Skin of Healthy Volunteers

#### 2.6.1. Volunteer Selection

The protocol for the in vivo study was submitted to the Bioethics Committee of the University of Patras, which determined that no special authorization was required for its execution. The volunteers were healthy men and women aged 20 to 55 years. Initially, they were asked questions regarding allergic reactions and general health status. Subsequently, the application areas (inner forearms) were examined. After being fully informed about the study’s nature and related procedures, they provided written consent. The participants did not suffer from any skin condition and were not taking medication that could affect the study’s results. Before the procedure began, volunteers remained for 20 min in a dedicated room where the temperature was maintained at 25 °C ± 2 °C.

#### 2.6.2. Evaluation of CUR Penetration Depth Using Tape-Stripping Technique on the Skin of Healthy Volunteers

The tape-stripping technique (TST) was applied to assess the depth of CUR penetration into the stratum corneum, following a modified procedure by Esposito et al. [27,28]. Initially, nine random 9 cm^2^ sites were defined on the inner surface of each volunteer’s forearm, and a 2 mg/cm^2^ dose of each nanocarrier was applied. Three sites per sample corresponded to different sampling times (30, 60, and 120 min). Uniform application of the samples was achieved through circular movements of a glove-wearing finger. Sample residues were removed with dry, soft paper 30 min after application. In total, five adhesive tapes (3.46 cm^2^/tape) were used per application site for sequential removal of SC layers. Each tape was pressed with a consistent force and held in place for a fixed time (10 consecutive passes with a cylindrical tool over the tape before removal).

Additionally, Jacobi and coworkers proposed an equation for correlating SC quantity with the number of removed adhesive tapes [29]. The relative thickness of the removed SC (percentage) was determined using Equation (6):

y = 107 − 111 × e^(−n/21)^, (6)

where y is the relative thickness of the SC, and n is the number of removed adhesive tapes.

After removal, the tapes were placed in three vials in the following order: 1st tape, 2nd and 3rd tapes, and 4th and 5th tapes. Each vial received 4 mL of EtOH for extraction, was tightly sealed, and stored overnight. The next day, samples were placed in an ultrasonic bath (30 kHz) for 20 min, centrifuged at 12,000 rpm for 6 min, and the supernatant of each sample was concentrated under vacuum to a final volume of 1 mL. CUR quantification was achieved using a calibration curve and UV–Vis spectrophotometry.

### 2.7. Statistical Analysis

Results are presented as the mean ± standard deviation (SD) of measurements from three independent replicates. The statistical significance of differences arising from comparing mean values was determined using a *t*-test (Microsoft Office 365 Excel 2016, Redmond, WA, USA), and/or ANOVA followed by Bonferroni’s multiple corrections was used for the assessments among the different experimental states (Graphpad Prism statistical software, version 10.4.1 (532), Graphpad Software, La Jolla, CA, USA). The significance level was set at *p* < 0.05.

## 3. Results

### 3.1. Physicochemical Characterization

#### 3.1.1. Measurement of Physicochemical Properties of Nanoparticles

Table 1 presents the results of the physicochemical characterization for each nanocarrier, measured one day after preparation.

The different fluidities of the empty nanocarriers (without CUR) seemed to affect their size, with the smallest size observed for NEs (122.60 nm ± 8.39 nm, *p* < 0.05) and the largest for SLNs (131.43 nm ± 9.94 nm, *p* < 0.05). The PdI remained around 0.2, indicating good homogeneity for all dispersions (*p* > 0.05). The ζ-potential was negative and near −60.00 mV for all nanocarriers (*p* > 0.05), suggesting good colloidal stability. The pH of all dispersions was acidic, averaging around 4.70 (*p* > 0.05).

Upon CUR loading in the nanocarriers, the physicochemical properties remained largely unaffected compared to their respective empty nanocarriers. Specifically, the size of all loaded nanocarriers was around 132.26 nm (*p* > 0.05), with the PdI near 0.2 (*p* > 0.05), maintaining good homogeneity. The ζ-potential remained negative, averaging around −61.00 mV (*p* > 0.05), preserving good colloidal stability. The dispersions’ pH remained acidic, near 4.50 (*p* > 0.05), compared to the reference CUR solution (CUR Sol.), which had an alkaline pH (Table 1).

#### 3.1.2. Morphological Characterization of Nanoparticles Using Transmission Electron Microscopy

**Figure 1:**
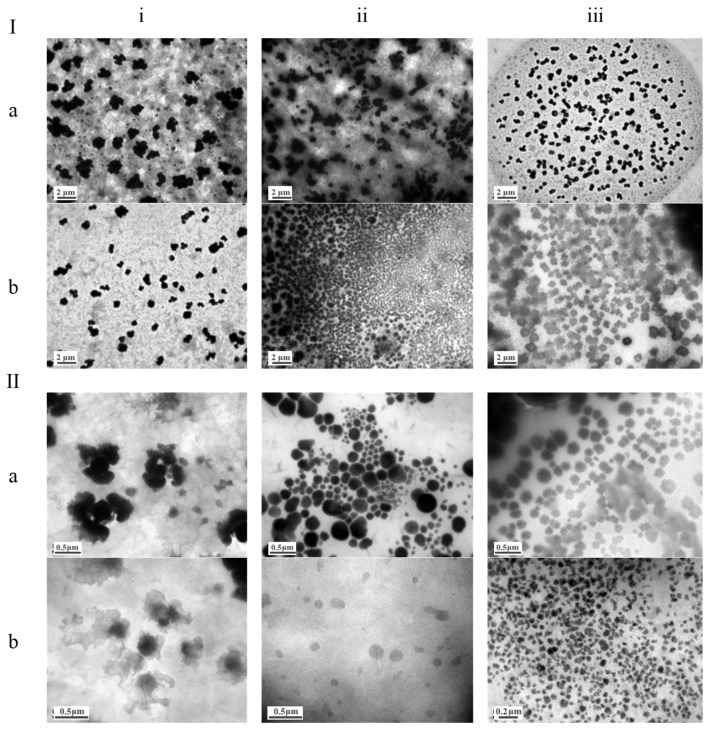
Transmission electron microscopy (TEM) images of lipid-based nanocarriers. Representative TEM images of (**i**) solid lipid nanoparticles (SLNs), (**ii**) nanostructured lipid carriers (NLCs), and (**iii**) nanoemulsions (NEs), either empty (**a**) or curcumin-loaded (**b**). The images were captured at various magnifications: ×6000 for empty and curcumin-loaded nanocarriers (**Ia**,**Ib**), ×30,000 for empty nanocarriers (**IIa**), and ×40,000/60,000 for curcumin-loaded nanocarriers (**IIbi**,**IIbii**,**IIbiii**). These images illustrate the morphological characteristics and structural differences between the nanocarriers, highlighting the effects of curcumin encapsulation on their appearance and organization.

The morphology of all nanoparticles was observed via transmission electron microscopy (TEM) one day after sample preparation. Negative staining (2% PTA solution) was applied. TEM images captured at different magnifications are shown in Figure 1. Particle size histograms were created by processing these images using the original ImageJ, displaying the size distribution of all nanoparticles (Figure S1) [21].

The TEM images showed a spherical morphology for all nanoparticles (Figure 1). The diameter of the empty nanoparticles ranged from 200–500 nm (left column in Figure S1), while the CUR-loaded nanoparticles ranged from 200–400 nm (right column in Figure S1). Therefore, CUR addition did not significantly affect the nanoparticles’ size, which generally appeared larger than the value determined by DLS, likely due to sample density and the resulting particle aggregation in the images.

#### 3.1.3. Quantitative Determination of CUR Loading and Encapsulation in Nanoparticles

The study on CUR loading in the nanocarriers was determined by UV–Vis spectrophotometry (Table 1). The actual loading of CUR in all nanoparticles was 1.49% ± 0.02% for NLC.CUR, 1.43% ± 0.02% for NE.CUR (*p* < 0.005), and 1.40% ± 0.01% for SLN.CUR (*p* < 0.005), while the theoretical loading averaged around 1.60% ± 0.01% (*p* > 0.05). The mean encapsulation efficiency was high for each nanocarrier, with NLC.CUR achieving the highest rate (93.71% ± 1.89%), and NE.CUR and SLN.CUR reaching 89.38% ± 1.73% (*p* < 0.005) and 87.61% ± 1.26% (*p* < 0.01), respectively. After the first day of preparation, the CUR content in the nanocarriers was found to be 0.0468% ± 0.0007% (*w*/*v*) for NLC.CUR, 0.0447% ± 0.0005% (*w*/*v*) for NE.CUR (*p* < 0.05), and 0.0437% ± 0.0001% (*w*/*v*) for SLN.CUR (*p* < 0.05).

### 3.2. Nanoparticle Stability Studies

#### 3.2.1. Centrifugation Test on Nanoparticles

All nanocarriers passed the centrifugation test at 5000 rpm for 30 min, as no phase separation was observed.

#### 3.2.2. Storage of Nanoparticles at 4 °C

**Figure 2:**
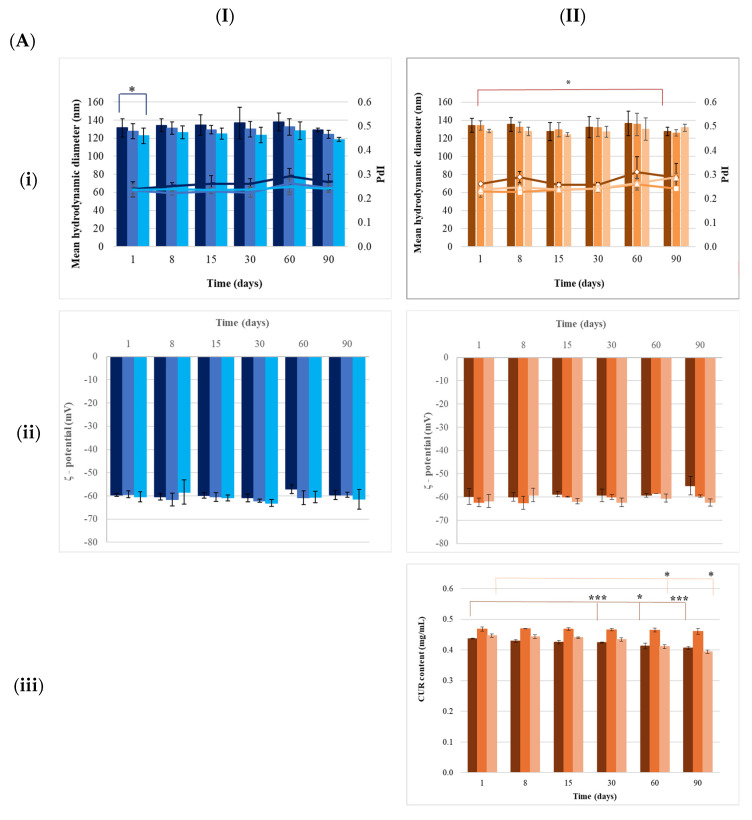

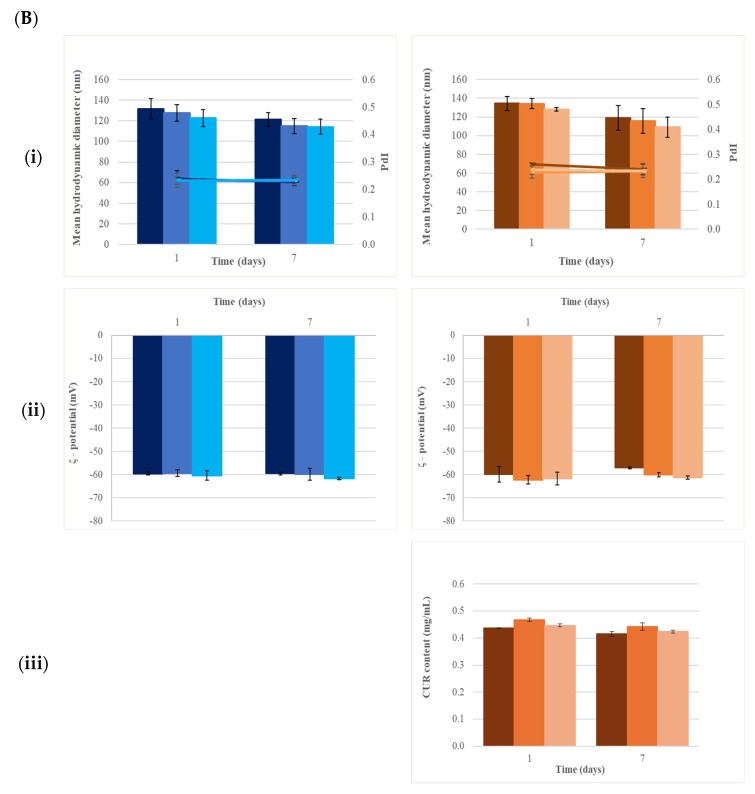
Stability study monitoring mean hydrodynamic diameter (column) and PdI (line) (**i**), ζ-potential (**ii**), and curcumin (CUR) content (**iii**) of empty nanocarriers (**I**): SLNs (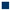), NLCs (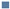), and NEs (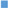), and curcumin-loaded nanocarriers (**II**): SLN.CUR (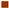), NLC.CUR (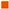), and NE.CUR (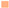), during storage for 90 days at 4 °C (**A**) and after accelerated aging (**B**). *: *p* < 0.05, ***: *p* < 0.005, n = 3.

All prepared nanoparticles maintained a stable size for 90 days at 4 °C (*p* > 0.05). The PdI remained stable, confirming good sample homogeneity. The ζ-potential, close to −60.00 mV, remained stable for all nanocarriers for at least 90 days (*p* > 0.05) (Table S2, Figure 2A).

With CUR addition, a slight decrease in the size of NLC.CUR was observed, approximately 6.23% on day 90 (*p* < 0.05, Figure 2(Iii)). However, the PdI remained stable near 0.2 over 90 days. The ζ-potential remained stable at around −61.00 mV for each nanocarrier (*p* > 0.05).

The CUR content only remained stable for at least 90 days in NLC.CUR (*p* > 0.05). In contrast, SLN.CUR showed a slight reduction of about 3% (*p* < 0.005), observed on day 30, with a similar decrease in NE.CUR on day 60, both decreasing by about 7% (*p* < 0.05) and 12% (*p* < 0.005) after 90 days of storage.

#### 3.2.3. Accelerated Aging Test on Nanoparticles

After three heating (45 °C) and cooling (25 °C) cycles, size, PdI, and ζ-potential measurements were taken for all nanocarriers. The results are presented in Table S3 and Figure 2B.

The accelerated aging test did not affect (*p* > 0.05) the size distribution or homogeneity of the empty and CUR-loaded nanocarriers. The ζ-potential remained stable regardless of the nanocarrier type (*p* > 0.05).

The CUR content in the nanoparticles during accelerated aging was determined by spectrophotometry after generating the corresponding standard curve. The results are presented in Table S3 and Figure 2(BIIiii).

The CUR content remained stable in all nanocarriers during accelerated aging (*p* > 0.05).

### 3.3. Antioxidant Activity of Nanoparticles

The antioxidant activity of the curcumin-loaded nanocarriers (SLN.CUR, NLC.CUR, NE.CUR) and free curcumin (CUR) was evaluated using two complementary methods: a DPPH assay, which measures radical scavenging activity, and a FRAP assay, which quantifies ferric-reducing antioxidant power. All samples contained 50 μg/mL of curcumin.

#### 3.3.1. Radical Scavenging Activity Assessed by the DPPH Method

The antioxidant activity of the CUR-loaded nanocarriers and free CUR was evaluated in comparison with ascorbic acid. Two standard curves showing the percentage of inhibition (% RSA) versus concentration (μg/mL) were created for ascorbic acid in aqueous (range: 70–1.25 μg/mL) and methanolic (range: 70–2.5 μg/mL) solutions, respectively, from which the required amounts of each sample were calculated to produce equivalent inhibition (Table S4).

The radical scavenging activity of the CUR-loaded nanocarriers (SLN.CUR, NLC.CUR, NE.CUR) and free CUR, expressed as % radical scavenging activity (%RSA), and ascorbic acid equivalents (μg/mL) are summarized in Table S5.

**Figure 3:**
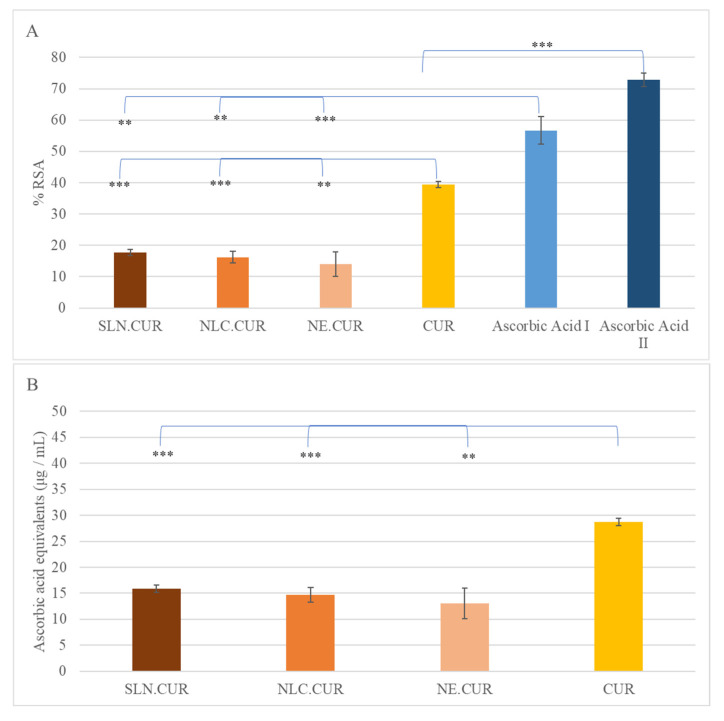
Antioxidant capacity ((**A**): %RSA, (**B**): ascorbic acid equivalents) of nanocarriers SLN.CUR (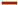), NLC.CUR (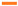), NE.CUR (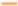), free CUR (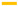), and ascorbic acid in aqueous (ascorbic I, 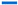) and methanolic (ascorbic II, 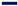) solutions. The initial concentration of CUR in all samples was 50 μg/mL. Results are expressed as a percentage inhibition (% RSA) of DPPH free radical (**: *p* < 0.01, ***: *p* < 0.005, n = 3).

Free curcumin exhibited significantly higher activity compared to the nanocarrier formulations (*p* < 0.005 for NE.CUR, *p* < 0.01 for SLN.CUR and NLC.CUR, Figure 3). Specifically, CUR demonstrated a value of %RSA of 39.40 ± 1.01%, corresponding to 28.74 ± 0.73 μg/mL ascorbic acid equivalents. In contrast, the %RSA values for SLN.CUR, NLC.CUR, and NE.CUR were 17.75 ± 0.94%, 16.23 ± 1.88%, and 14.03 ± 3.92%, respectively. Similarly, their ascorbic acid equivalents were significantly lower, ranging from 13.04 ± 2.94 μg/mL (NE.CUR) to 15.83 ± 0.71 μg/mL (SLN.CUR).

CUR encapsulation reduced this capacity by approximately half compared to free CUR (*p* < 0.005 for SLN.CUR and NLC.CUR and *p* < 0.01 for NE.CUR, Figure 3B).

#### 3.3.2. Ferric Ion Reducing Power Measured by the FRAP Method

The antioxidant activity of the nanocarriers was evaluated in comparison with ascorbic acid. Two standard curves, showing absorbance at 595 nm versus concentration (μg/mL), were created for ascorbic acid in aqueous and methanolic solutions, from which the required amounts of each sample were calculated to achieve equivalent antioxidant effects. Table S6 lists the concentrations of aqueous and methanolic ascorbic acid solutions used to create the standard curves.

**Figure 4:**
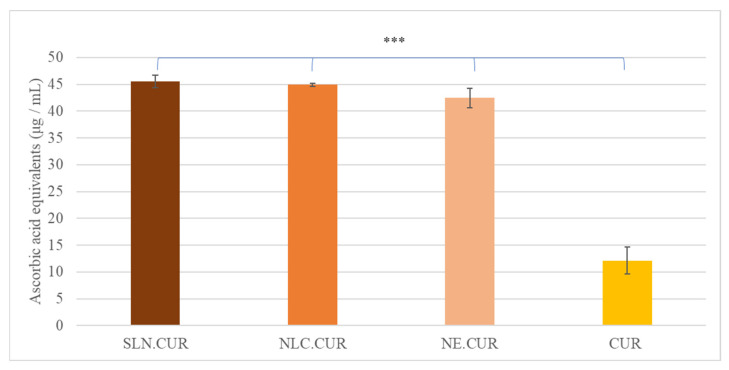
Antioxidant activity of SLN.CUR (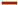), NLC.CUR (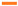), NE.CUR (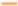), and free CUR (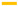) as determined by the FRAP method. Results are expressed as ascorbic acid equivalents (μg/mL), (***: *p* < 0.005).

Figure 4 and Table S7 present the ferric-reducing antioxidant power of all samples, expressed as ascorbic acid equivalents (μg/mL).

The curcumin-loaded nanocarriers exhibited significantly higher antioxidant activity compared to free curcumin (*p* < 0.005). The ascorbic acid equivalents for SLN.CUR, NLC.CUR, and NE.CUR were 45.52 ± 1.20, 44.93 ± 0.30, and 42.44 ± 1.77 μg/mL, respectively, while free curcumin exhibited only 12.13 ± 2.47 μg/mL (Figure 4).

### 3.4. Cytotoxicity and Gene Expression

#### 3.4.1. In Vitro Cell Viability Assay

An MTT assay was used to assess the cell viability of the NHDF cells under the treatment of the different samples in different concentrations (0.05–1.00 μg/mL). Figure S2 presents the main outcomes of this analysis. No cytotoxicity was observed for the NHDF cells treated with different concentrations of samples of nanodispersions (0.05–1 μg/mL) (Figure S2). However, for the transcriptomic analysis, we opted for a concentration of 1%, as this represents the optimal concentration of nanodispersions in cosmetic formulations.

#### 3.4.2. Gene Expression Analysis

**Figure 5:**
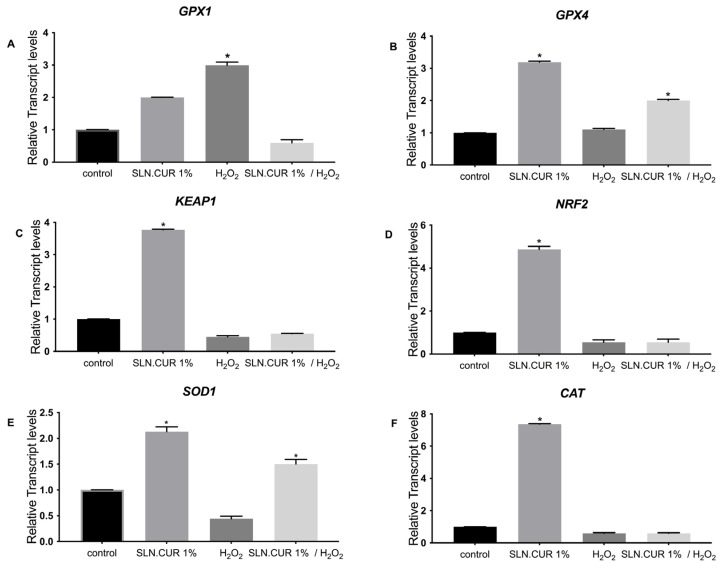
Relative expression of (**A**) *GPX1*, (**B**) *GPX4*, (**C**) *KEAP1* (**D**) *NRF2*, (**E**) *SOD1*, and (**F**) *CAT* in control (untreated NHDF), NHDF cells treated with SLN.CUR (1 μg/mL) (SLN.CUR 1%), NHDF cells treated with H_2_O_2_ 0.5 mM (H_2_O_2_), and NHDF cells treated with SLN.CUR 1% (1 μg/mL) and H_2_O_2_ (0.5 mM) (SLN.CUR 1%/H_2_O_2_). Transcript expression levels were obtained by qPCR, and the means of ACTB and *GADPH* were used as internal reference genes. The results are presented as a fold change ± SD with respect to the control and represent the mean ± SEM of three independent experiments. * *p* < 0.05 significantly different from control using one-way ANOVA.

**Figure 6:**
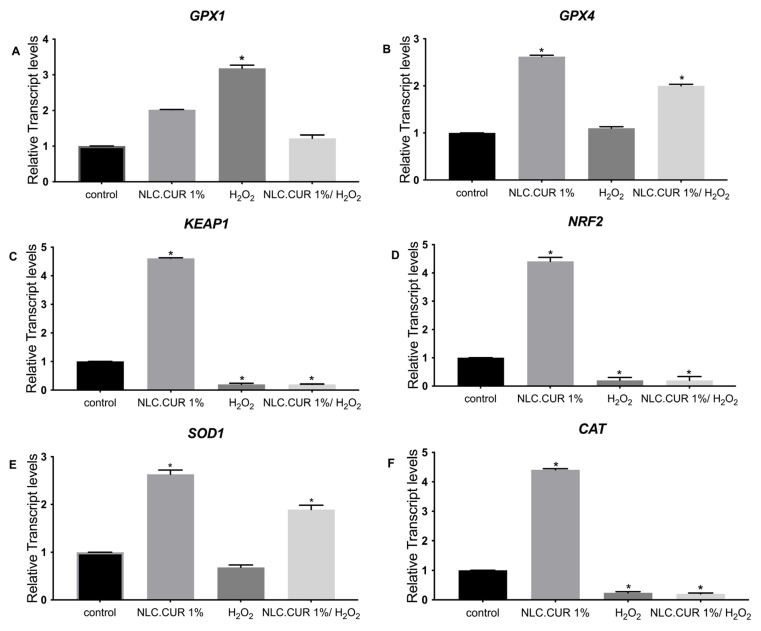
Relative expression of (**A**) *GPX1*, (**B**) *GPX4*, (**C**) *KEAP1* (**D**) *NRF2*, (**E**) *SOD1*, and (**F**) *CAT* in control (untreated NHDF), NHDF cells treated with NLC.CUR 1% (1 μg/mL) (NLC.CUR 1%), NHDF cells treated with H_2_O_2_ 0.5 mM (H_2_O_2_), and NHDF cells treated with NLC.CUR 1% and H_2_O_2_ (0.5 mM) (NLC.CUR 1%/H_2_O_2_). Transcript expression levels were obtained by qPCR, and the means of *ACTB* and *GADPH* were used as internal reference genes. The results are presented as a fold change ± SD with respect to the control and represent the mean ± SEM of three independent experiments. * *p* < 0.05 significantly different from control using one-way ANOVA.

**Figure 7:**
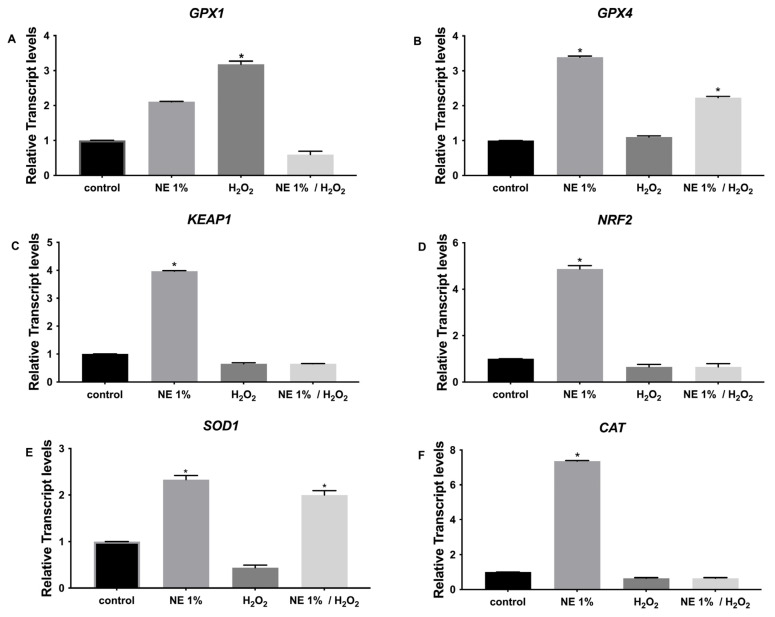
Relative expression of (**A**) *GPX1*, (**B**) *GPX4*, (**C**) *KEAP1* (**D**) *NRF2*, (**E**) *SOD1*, and (**F**) *CAT* in control (untreated NHDF), NHDF cells treated with NE.CUR 1% (1 μg/mL) (NE.CUR 1%), NHDF cells treated with H_2_O_2_ 0.5 mM (H_2_O_2_), and NHDF cells treated with NE.CUR 1% and H_2_O_2_ (0.5 mM) (NE.CUR 1%/H_2_O_2_). Transcript expression levels were obtained by qPCR, and the means of *ACTB* and *GADPH* were used as internal reference genes. The results are presented as a fold change ± SD with respect to the control and represent the mean ± SEM of three independent experiments. * *p* < 0.05 significantly different from control using one-way ANOVA.

**Figure 8:**
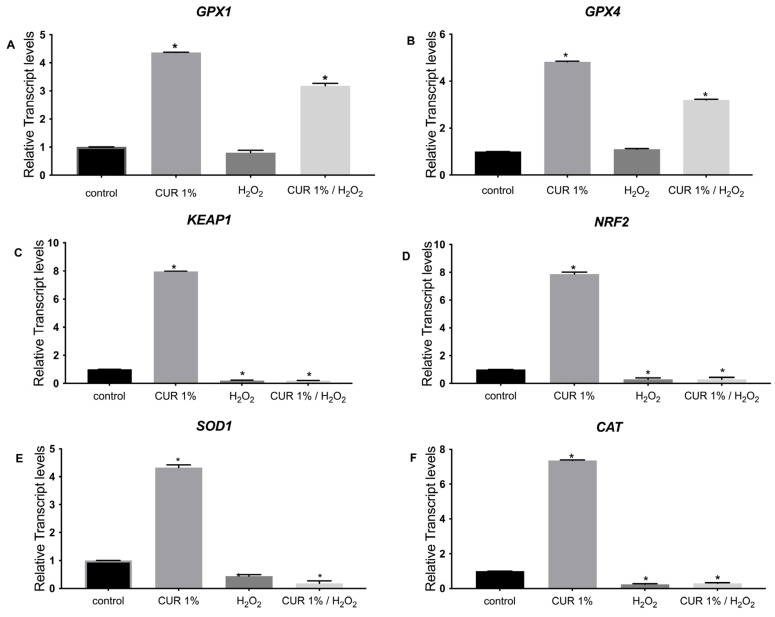
Relative expression of (**A**) *GPX1*, (**B**) *GPX4*, (**C**) *KEAP1* (**D**) *NRF2*, (**E**) *SOD1*, and (**F**) *CAT* in control (untreated NHDF), NHDF cells treated with CUR 1% (1 μg/mL) (CUR 1%), NHDF cells treated with H_2_O_2_ 0.5 mM (H_2_O_2_), and NHDF cells treated with CUR 1% and H_2_O_2_ (0.5 mM) (CUR 1%/H_2_O_2_). Transcript expression levels were obtained by qPCR, and the means of *ACTB* and *GADPH* were used as internal reference genes. The results are presented as a fold change ± SD with respect to the control and represent the mean ± SEM of three independent experiments. * *p* < 0.05 significantly different from control using one-way ANOVA.

According to Figure 5, we observed that in the case of CUR.SLN (1%), the expression of *GPX1* was upregulated only under oxidative stress independently of the presence of SLN, while the expression of *KEAP1*, *NRF2*, and *CAT* was upregulated only with the presence of CUR.SLN compared to the control (*p* < 0.05, Figure 5A,C,D,F). In addition, the expression of *GPX4* and *SOD1* was upregulated with the presence of CUR.SLN with or without oxidative stress compared to the control (*p* < 0.05, Figure 5B,E). In the case of NLC.CUR, the expression of *GPX1* and *GPX4* was induced with or without oxidative stress compared to the control (*p* < 0.05, Figure 6A,B). The expression of *SOD1*, *KEAP1*, *CAT*, and *NRF2* was upregulated with the presence of CUR.NLC at 1%, while it was downregulated under oxidative stress as compared to the control (*p* < 0.05, Figure 6C–F). The same gene profile was observed in the samples CUR-NE (1%) and CUR (1%), respectively (Figure 7 and Figure 8).

### 3.5. Effect of Nanoparticles on SC Permeability

The effect of the three nanocarriers on CUR penetration depth was examined after application on the skin of healthy volunteers. The amount of CUR was then determined for each tape removed at specific intervals (30, 60, 120 min). The application of five adhesive tapes corresponded to approximately 20% SC removal [29].

The amount of CUR detected on each tape at each time point, as well as the total CUR amount in the SC, is summarized in Table S7.

**Figure 9:**
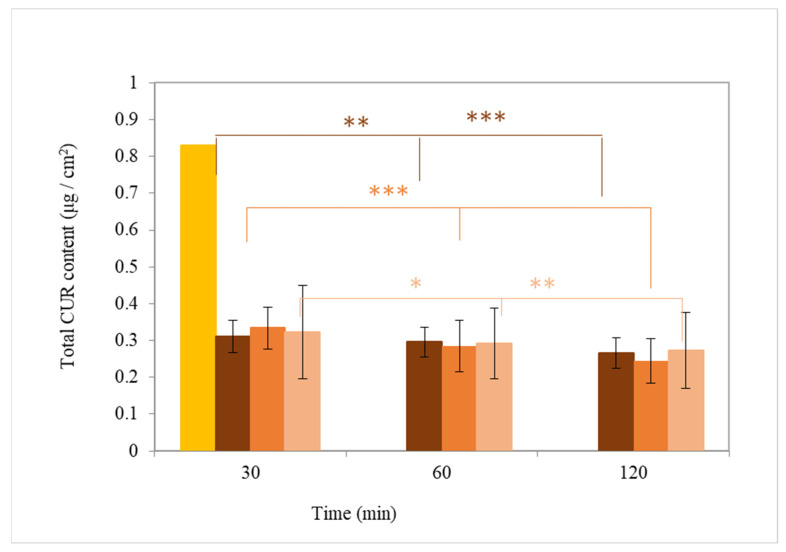
Quantification of total CUR (μg/cm^2^) incorporated into the SC of volunteers after 30, 60, and 120 min of application of the nanocarriers: SLN.CUR (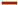), NLC.CUR (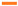), and NE.CUR (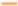) compared to the initial quantity of CUR (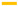) (*: *p* < 0.05, **: *p* < 0.01, ***: *p* < 0.005).

The initial amount of CUR applied to the skin at each time point was 0.83 µg/cm^2^. However, after 30 min, the total amount of CUR detected by analysis of the films was, for all nanocarriers, smaller by 61% (*p* < 0.005, Figure 9).

**Figure 10:**
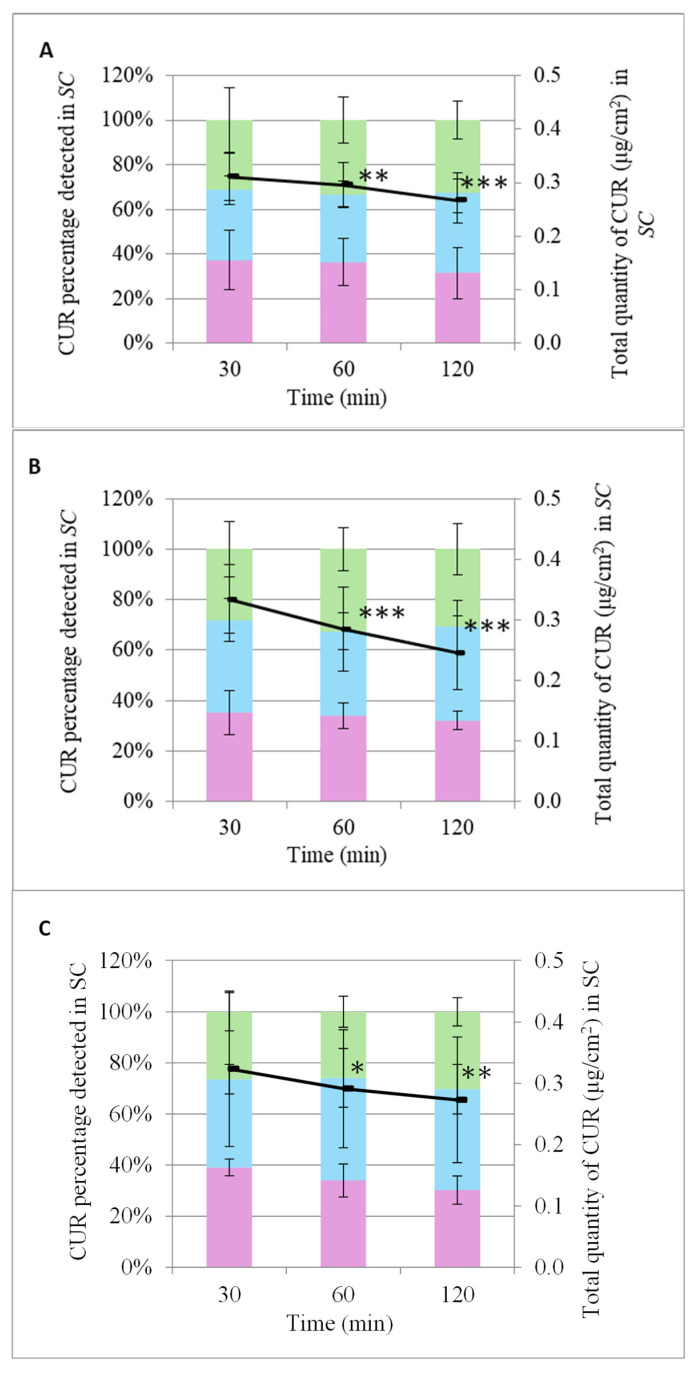
The incorporation of total quantity of CUR (line) in the SC and the distribution at different depths (bars) of SC of volunteers after the application of SLN.CUR (**A**), NLC.CUR (**B**), and NE.CUR (**C**) and tape stripping. Tapes 1: 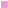, 2 + 3: 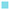, 4 + 5: 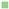. *: *p* < 0.05, **: *p* < 0.01, ***: *p* < 0.005.

The encapsulated CUR in SLN.CUR was detected deeper in the SC from the first 30 min and was evenly distributed (~33%, *p* > 0.05) across all layers of the SC up to 60 min. However, a reduction in the total amount of CUR was observed at 60 min, which continued progressively until 120 min (Figure 10). After 120 min, the percentage detected in the upper layer decreased (31.41% ± 11.39%, *p* < 0.05), while the quantity in the intermediate layer increased (36.05% ± 8.85%, *p* < 0.05), and the deeper layer maintained the same levels (32.54% ± 8.49%, *p* > 0.05). These results indicate the rapid penetration of CUR into the SC when it was incorporated into SLNs (Table S8, Figure 10A).

The encapsulated CUR in NLC.CUR was detected deeper in the SC from the first 30 min, and it was evenly distributed (~33%, *p* > 0.05) across all depths of the SC during the 120 min. However, a reduction in the total amount of CUR was observed at 60 min, which continued progressively until 120 min (Figure 10). These results also show the increased penetration of CUR into the SC when incorporated into NLCs (Table S8, Figure 10B).

The encapsulated CUR in NE.CUR was distributed across all layers of the SC from the first 30 min, with the largest percentage (39.08% ± 3.30%, *p* < 0.05) concentrated in the upper layer and a smaller amount in the deeper layer (26.40% ± 7.45%, *p* < 0.005). A reduction in the total amount of CUR was also observed over time. After 60 min, a significant percentage of the CUR was also found in the intermediate layer (40.14% ± 11.48%, *p* < 0.05), while the percentage corresponding to the deeper layer remained stable (25.86% ± 6.08%, *p* > 0.05). At 120 min, an equal distribution of the quantity was observed across all layers due to the slight increase in the percentage occurring in the deeper layer (30.28% ± 5.53%, *p* < 0.05). These results show that incorporating CUR into NEs led to a significantly increased penetration into the SC, which, however, appeared to be achieved at a slower rate compared to the SLNs and NLCs (Table S8, Figure 10C).

## 4. Discussion

Curcumin is an interesting molecule as it is involved in the treatment of several diseases, particularly chronic ones, ranging from cancer to skin diseases [30,31]. This study explored the potential of lipid-based nanocarriers—solid lipid nanoparticles (SLNs), nanostructured lipid carriers (NLCs), and nanoemulsions (NEs)—to enhance curcumin’s bioactivity for mitigating oxidative stress in human skin cells. Given the challenges associated with curcumin’s solubility, stability, and bioavailability, these nanocarriers offer a promising means to improve its therapeutic efficacy, particularly in topical formulations targeting skin disorders [30,31]. Our results show that encapsulating curcumin in SLN, NLC, and NE structures provides substantial advantages in terms of stability, antioxidant activity, and cellular penetration, ultimately underscoring the utility of these carriers for oxidative-stress-related skin applications.

The physicochemical characterization of the three lipid nanocarriers confirmed their suitability for encapsulating curcumin. The mean hydrodynamic diameter of the empty nanocarriers was dependent on the type of nanocarrier, with NEs exhibiting the smallest size (122.60 ± 8.39 nm, *p* < 0.05) and SLNs the largest (131.43 ± 9.94 nm, *p* < 0.05). Although statistically significant, these differences were relatively small. Upon curcumin loading, the differences in size became statistically non-significant, and the mean hydrodynamic diameter was approximately 132.26 nm (*p* > 0.05) for all formulations. The polydispersity index (PdI), an indicator of dispersion homogeneity, remained consistent at around 0.2 for both the empty and curcumin-loaded nanocarriers (*p* > 0.05), indicating good sample uniformity.

Compared to the literature, our formulations exhibited smaller particle sizes. For example, Araújo et al. reported mean sizes of approximately 190 nm and 219 nm for curcumin-loaded NEs and NLCs, respectively [32]. Espinosa-Olivares et al. reported a size range of 111 to 214 nm for curcuminoid-loaded NLC formulations, depending on the composition [33]. Similarly, Aydin et al. observed that curcumin-loaded SLNs had particle sizes ranging from 203.8 to 353.8 nm [34]. These discrepancies could be attributed to differences in formulation methods, lipid compositions, and emulsifier types. Despite variations in size, all these studies, including ours, reported PdI values below 0.3, confirming satisfactory homogeneity of the dispersions.

The ζ-potential of the nanocarriers, a measure of colloidal stability, was consistently negative and near −60.00 mV for both the empty and curcumin-loaded formulations (*p* > 0.05). This indicates excellent colloidal stability, which is essential for maintaining the structural integrity of the nanocarriers over time. In contrast, Araújo et al. reported ζ-potentials of approximately −20 mV for curcumin-loaded NEs and −25 mV for NLCs, which may reflect differences in formulation components such as surfactants or lipids [32]. Espinosa-Olivares et al. observed ζ-potentials ranging from −4.1 mV to −15.5 mV for curcumin-loaded NLCs, depending on the ingredients used, while Aydin et al. reported a ζ-potential of approximately −37.8 mV for curcumin-loaded SLNs [33,34]. The higher magnitude of the ζ-potential observed in our study suggests improved colloidal stability compared to these formulations.

The pH of all the dispersions was acidic, averaging around 4.70 (*p* > 0.05), which aligns with the stability requirements of curcumin and the physiological pH range of healthy skin. This acidic environment helps to maintain curcumin’s stability and ensures the formulations are suitable for topical application without compromising skin barrier integrity. Each formulation exhibited distinct features that influenced its stability, release profile, and interaction with skin cells. SLNs, characterized by a more rigid structure, maintained high stability and sustained release, which is advantageous for prolonged therapeutic effects [19,35]. NLCs, owing to their mixed solid–liquid lipid composition, demonstrated a higher drug-loading capacity and improved flexibility, enhancing curcumin’s skin penetration potential [16]. NEs, with their fine droplet size and high surface area, allowed for rapid curcumin release and facilitated immediate antioxidant effects [12,36]. Together, these nanocarriers provide a diverse toolkit for modulating curcumin’s delivery depending on the therapeutic needs of specific skin conditions.

The stability studies conducted in this work demonstrated that all three nanocarriers —SLNs, NLCs, and NEs—maintained their structural integrity and curcumin content over time, with only slight variations in particle size and ζ-potential. Among the formulations, NLCs showed the highest stability, with minimal changes in physicochemical properties over 90 days of storage, highlighting their suitability for long-term formulations. SLNs and NEs, while slightly more variable, still demonstrated acceptable stability, with only minor reductions in curcumin content, particularly in the NE formulations. These results are critical for developing reliable skincare products, as they confirm that curcumin-loaded nanocarriers can withstand storage without significant degradation or loss of bioactivity. Such stability is essential for applications where extended shelf-life and consistent efficacy are necessary.

When compared to the literature, our findings align with and expand upon previous reports. Espinosa-Olivares et al. observed fluctuations in the mean size of curcumin-loaded NLCs after storage for three months at 5 °C and 25 °C, emphasizing the importance of ingredient selection on nanocarrier stability [33]. Similarly, Araújo et al. reported small increases in size for NEs across all storage temperatures, while NLCs showed greater size variations over time, although the PdI and ζ-potential remained largely unchanged for both formulations [32]. These results are consistent with the trends observed in our study, where NLCs demonstrated superior stability, further supporting their robustness for long-term use.

Aydin et al. evaluated curcumin-loaded SLNs and noted no statistically significant variations in the particle size, ζ-potential, or PdI over a 60-day storage period at 5 ± 3 °C. They attributed this stability to the high zeta potential (−37.8 ± 1.4 mV) of the optimized SLN formulation, which exceeded the −30 mV threshold commonly associated with stable colloidal systems [34]. Our study similarly found that SLNs retained their structural and colloidal stability over time, with ζ-potential values consistently supporting their stability under specified storage conditions.

The antioxidant activity of the curcumin-loaded nanocarriers was evaluated using DPPH and FRAP assays, which assess antioxidant mechanisms differently. The DPPH assay measures free radical scavenging activity via electron transfer, while the FRAP assay quantifies the ability to reduce ferric ions, reflecting the total reducing power. Variations in the results between these assays may arise due to differences in the mechanisms they measure. For instance, the fluidity of lipid nanocarriers could influence the accessibility of curcumin to free radicals (DPPH assay) versus its interaction with ferric ions (FRAP assay). This dual assessment highlights the importance of using complementary methods to fully characterize the antioxidant potential of nanocarrier systems.

The antioxidant capacity, expressed as free radical scavenging activity, in the DPPH assay revealed that encapsulation of CUR reduced the scavenging activity by half for all nanocarriers.

The lower radical scavenging activity of the nanocarrier formulations can be attributed to the incomplete release of curcumin during the assay. While the methanol–water mixture used in the DPPH assay might have partially disrupted the nanocarriers, the release of curcumin was not instantaneous, leaving some of it inaccessible for direct interaction with the DPPH radical.

Encapsulation may hinder the release of protons from the molecular structure of CUR, which are necessary for binding free radicals [37]. The encapsulation of CUR in an NE nanocarrier showed a decrease in scavenging capacity at a low concentration (<15 ppm) according to Saari and colleagues, while a slight reduction (~6%) in antioxidant activity was also reported by Sari and colleagues after CUR encapsulation in NEs compared to free CUR [38,39]. Furthermore, interference from the lipid matrix may have contributed to the reduced activity of the encapsulated formulations [23]

The ferric-reducing antioxidant power of the samples, expressed as ascorbic acid equivalents, showed an opposite trend. The enhanced antioxidant activity of the nanocarriers in the FRAP assay can be attributed to multiple factors. At the acidic pH (3.6) of the FRAP assay, free curcumin exhibited poor solubility and reduced stability, limiting its ability to participate effectively in the redox reaction [40,41]. Conversely, the encapsulation of curcumin within the nanocarriers improved its solubility and protected it from degradation, thereby enhancing its reducing power. Additionally, the lipid matrix of the nanocarriers may have contributed synergistically to the overall ferric-reducing capacity.

The observed differences in antioxidant activity between the DPPH and FRAP assays highlight the influence of assay conditions and curcumin formulations on the results. The DPPH assay favors free curcumin due to its immediate accessibility for radical scavenging, whereas the FRAP assay underscores the advantages of nanocarriers in enhancing curcumin’s solubility, stability, and sustained release under acidic conditions. These findings emphasize the importance of selecting appropriate antioxidant assays to evaluate the functionality of curcumin and its formulations.

The cytotoxicity assays further validated the safety profile of the curcumin-loaded nanocarriers, with all formulations demonstrating minimal cytotoxicity in human dermal fibroblasts. Cell viability remained above 80% across all nanocarrier-treated groups, underscoring their biocompatibility and suitability for skin applications. This outcome is particularly encouraging, as it suggests that these formulations can deliver effective antioxidant protection without compromising cell health. It also suggests that the lipid composition of SLNs, NLCs, and NEs is well-tolerated by human skin cells, making them favorable candidates for further development in dermatological treatments. Previous reports support that increased cell viability is associated with higher levels of mitochondrial activity as well as cell proliferation [42,43]. In addition, previous studies confirm the lack of cytotoxicity of NE, SLN, and NLC lipid nanocarriers, as well as curcumin [12].

To gain a deeper understanding of the molecular mechanisms by which encapsulated curcumin combats oxidative stress, we examined the expression of key genes involved in cellular antioxidant pathways. Our findings indicate that curcumin encapsulated within NE, SLN, and NLC lipid nanocarriers modulates the transcription of genes critical to antioxidant defense.

An important aspect of this study was the gene expression analysis of key antioxidant markers. Specifically, we analyzed the expression of *GPX1*, *GPX4*, *SOD1*, *CAT*, *KEAP1*, and *NRF2*, which are widely recognized as essential antioxidant markers [43,44,45,46,47]. Previous studies have reported the upregulation of these genes under oxidative stress conditions, highlighting their roles in cellular protection [43,48,49]. In this study, we observed a significant upregulation of *GPX4* and *SOD1* in response to curcumin encapsulated in NEs, SLNs, and NLCs, both in the presence and absence of oxidative stress, compared to controls. This result suggests a robust antioxidant protective effect provided by the lipid nanocarriers.

Notably, free curcumin (without lipid encapsulation) also induced the expression of *GPX1* and *GPX4*, indicating a *GPX*-dependent antioxidant response to oxidative stress. However, encapsulation within the lipid nanocarriers further enhanced the expression of *SOD1*, a gene crucial to cellular antioxidant defense, underscoring the added benefit of these carriers in supporting curcumin’s protective effects. Moreover, curcumin encapsulated within NE, SLN, and NLC nanocarriers increased *KEAP1*, *NRF2*, and *CAT* expression under non-stress conditions, indicating a preemptive antioxidant action that fortifies cells against potential oxidative damage. Similarly, unencapsulated curcumin also upregulated *SOD1*, *KEAP1*, *CAT*, and *NRF2* in the absence of oxidative stress, reinforcing its innate antioxidant properties.

Overall, each formulation demonstrated a distinct antioxidant profile, providing protective effects both with and without oxidative stress, thereby confirming the potential of curcumin-loaded lipid nanocarriers in enhancing cellular resilience and antioxidant capacity.

The differential expression patterns observed with each nanocarrier type reveal the unique interactions between curcumin, the carrier lipid matrix, and the cellular environment. SLNs and NLCs, with their distinct lipid compositions, may influence curcumin’s activity by modulating its release rate and interaction with cell membranes [19]. For instance, the rigid structure of SLNs may facilitate a steady release that induces gradual antioxidant gene activation, which could be beneficial for chronic oxidative stress conditions such as photoaging. In contrast, the fluid structure of NLCs and NEs promotes faster curcumin release, leading to a more immediate but transient activation of antioxidant genes, which could be useful for acute oxidative stress conditions, such as sunburn. These distinctions indicate that by selecting the appropriate lipid nanocarrier, it is possible to tailor curcumin’s therapeutic effects to meet the specific needs of different oxidative-stress-related skin disorders.

The penetration of CUR from the three nanocarriers (SLNs, NLCs, and NEs) was analyzed in vivo with respect to the total amount reaching 20% of the stratum corneum (SC) in the skin of volunteers. The results indicated that the fluidity of the nanoparticle matrix did not significantly affect their penetration ability within the SC during the 120 min application period. In a similar in vivo study, the total amount of CUR that penetrated the SC was generally higher for NLC systems than for NE systems [50]. The lower total amount of CUR detected in the final analysis may be due to the sensitivity of the analytical method, CUR’s rapid metabolism, and other limitations associated with this biomolecule, but it was primarily due to the removal of any CUR remaining on the SC surface before applying the tapes. However, further penetration of CUR beyond the initially studied 20% depth of the SC is equally possible.

The results of our previous work on an in vitro penetration study using Franz cells, combined with those from the in vivo TST test of the present work, are quite interesting, as they demonstrated that the synthesized curcumin nanocarriers, despite their small size (<140 nm), did not penetrate beyond a few layers of the SC, which is also related to the idea of slow release from these nanosystems [19]. These findings confirm the theory that nanotechnology can modify a substance’s penetration ability and control its release, increasing its residence time on the skin surface [19,51]. These results are highly encouraging, as they highlight the safe use of nanoparticles in cosmetic products, as they do not enter systemic circulation.

While our study demonstrates the enhanced skin penetration of curcumin-loaded nanocarriers, it is important to acknowledge the limitations of the in vivo penetration model. Factors such as curcumin metabolism and clearance may influence its bioavailability and therapeutic efficacy, particularly in dynamic biological systems. Additionally, the model did not account for variables such as repeated application, the effects of enzymatic degradation, or interactions with other skin components. Future studies should focus on addressing these limitations through more advanced models and incorporating strategies to stabilize curcumin post-penetration, ensuring sustained therapeutic effects.

A practical implication of these findings is the potential for these nanocarriers to be used in different skincare formulations depending on the specific therapeutic goals. The targeted skin conditions, including psoriasis and acne, are characterized by elevated levels of oxidative stress and inflammation. Curcumin-loaded nanocarriers have the potential to mitigate these effects by delivering curcumin more efficiently to the skin. SLNs, with their sustained release properties, may be particularly suitable for chronic conditions such as psoriasis, where prolonged antioxidant and anti-inflammatory activity is beneficial. In contrast, the enhanced flexibility and deeper skin penetration of NLCs and NEs make them promising candidates for treating acute conditions such as acne, where rapid ROS scavenging and inflammation reduction are critical. An important practical takeaway from these findings is the adaptability of these nanocarriers for integration into skincare formulations designed to meet specific therapeutic objectives. SLNs, with their ability to provide sustained antioxidant delivery, could be ideal for long-term applications such as anti-aging creams. NLCs, offering enhanced skin penetration and structural flexibility, are better suited for treatments requiring deeper dermal impact, such as those targeting inflammatory skin conditions. NEs, with their rapid release capabilities, may be optimal for products designed to provide immediate relief, such as after-sun lotions or treatments for acute irritation. These nanocarriers offer versatility in formulation design, making them adaptable to various skin health applications.

Overall, the encapsulation of curcumin in lipid nanocarriers marks a significant advancement in enhancing its therapeutic potential for oxidative-stress-related skin treatments. By improving curcumin’s stability, bioavailability, and antioxidant activity, these nanocarriers overcome key limitations associated with traditional curcumin formulations. The ability to modulate curcumin’s release and gene activation profile through carrier selection offers an opportunity to develop highly targeted skin therapies. Future studies could explore the synergistic effects of combining curcumin with other natural antioxidants or therapeutic agents within these lipid nanocarriers, potentially broadening their applicability and efficacy.

## 5. Conclusions

This study demonstrates that lipid nanocarriers—solid lipid nanoparticles (SLNs), nanostructured lipid carriers (NLCs), and nanoemulsions (NEs)—significantly enhance the stability and antioxidant activity of curcumin, making them highly suitable for combating oxidative stress in skin applications. Each nanocarrier type offers unique advantages in terms of release profile, skin penetration, and antioxidant gene activation, providing valuable insights into how these carriers can be used to tailor curcumin delivery based on specific therapeutic needs. SLNs proved to be the most stable nanocarrier, with a sustained release profile ideal for prolonged skin protection, while NLCs and NEs offered enhanced flexibility, rapid release, and improved dermal penetration. Antioxidant gene expression analysis revealed that these carriers not only deliver curcumin effectively but also modulate key antioxidant genes, such as *GPX1*, *GPX4*, *SOD1*, *KEAP1*, and *NRF2*, reinforcing the potential for targeted therapeutic action at a molecular level. With demonstrated biocompatibility and low cytotoxicity, these formulations hold promise for treating oxidative-stress-related skin disorders. Future studies should focus on optimizing these carriers for clinical applications and exploring their potential in commercial skincare products.

## Figures and Tables

**Table 1:** Key physicochemical properties of nanocarriers on day 1 after preparation.

Sample	Hydrodynamic Diameter (nm)	PdI	ζ-Potential (mV)	pH	Theoretical Loading (%)	Actual Loading (%)	Encapsulation Efficiency (%)
SLNs	131.43 ± 9.94	0.24 ± 0.03	−59.58 ± 0.55	4.73 ± 0.07			
NLCs	127.71 ± 8.19	0.23 ± 0.01	−59.36 ±1.48	4.59 ± 0.05			
NEs	122.60 ± 8.39	0.23 ± 0.03	−60.38 ± 2.09	4.80 ±0.13			
SLN.CUR	134.32 ± 7.66	0.26 ± 0.00	−59.79 ± 3.35	4.25 ± 0.07	1.60 ± 0.00	1.40 ± 0.01	87.61 ± 1.26
NLC.CUR	134.23 ± 5.32	0.23 ± 0.02	−62.22 ± 1.82	4.64 ± 0.03	1.59 ± 0.01	1.49 ± 0.02	93.71 ± 1.89
NE.CUR	128.23 ± 1.97	0.24 ± 0.02	−61.70 ± 2.79	4.73 ± 0.01	1.60 ± 0.01	1.43 ± 0.02	89.38 ± 1.73
CUR Sol.	-	-	-	7.49 ± 0.03			

## Data Availability

Data are available upon request.

## Supplementary Materials

The following supporting information can be downloaded at: https://www.mdpi.com/article/10.3390/pharmaceutics17020144/s1, Table S1. Gene name. Accesion No. Kegg pathway. Primmers. Table S2. Stability study of the physicochemical characteristics of the nanocarriers during storage at 4 °C. Table S3. Changes in physicochemical characteristics of nanocarriers during the accelerated aging test; Table S4. Concentrations of aqueous (A) and methanolic (B) ascorbic acid solutions and corresponding DPPH radical inhibition percentage; Table S5. Antioxidant activity of CUR-loaded nanocarriers (SLNs, NLCs, and NEs) and free CUR expressed as DPPH radical inhibition percentage (% RSA) and ascorbic acid equivalents (μg/mL); Table S6. Concentrations of aqueous (A) and methanolic (B) ascorbic acid solutions and corresponding absorbance at 595 nm for standard curve; Table S7. Antioxidant activity of nanocarriers SLN.CUR, NLC.CUR, and NE.CUR and free CUR expressed as ascorbic acid equivalents (μg/mL); Table S8. Quantitative determination of CUR in nanocarriers after tape stripping from the skin of healthy volunteers; Figure S1. Size distribution for empty nanoparticles (left) and those loaded with CUR (right), as derived from the analysis of TEM microscope images; Figure S2. Cell viability assessments for control, curcumin, NEs, NLCs, and SLNs. The results are presented as a percentage ± SD with respect to the control and represent the mean ± SEM of three independent experiments.
