# Supplementary material for: Curcumin-Loaded Lipid Nanocarriers: A Targeted Approach for Combating Oxidative Stress in Skin Applications
Source: Pharmaceutics. 2025 Jan 21;17(2):144. doi: 10.3390/pharmaceutics17020144 (PMC11859734; doi:10.3390/pharmaceutics17020144)
Supplement: Supplementary file 1 [file pharmaceutics-17-00144-s001.zip › pharmaceutics-3401227-supplementary.pdf]

Curcumin-Loaded Lipid Nanocarriers: A Targeted Approach for Combating Oxidative Stress in Skin Applications

Aggeliki Liakopoulou [1], Sophia Letsiou [2], Konstantinos Avgoustakis [1], George Patrinos [2], Fotini N. Lamari [3], and Sophia Hatziantoniou [1,*]

Supplement Materials

2.5.3. RNA isolation, cDNA synthesis and PCR analysis.
Table S1. Gene name. Accesion No. Kegg pathway. Primmers

Gene Symbol	Gene Name	Accession No	KEGG pathway	Primmer F (5'-3')	Primmer R (5'-3')
ACTB	actin. beta	NM_001101.3	hsa04810	CTGTCCACCTTC CAGCAGATGT	AGCATTTGCGG TGGACGAT
GAPDH	glyceraldehyde-3-phosphate dehydrogenase	NM_001256799.2	hsa01200	TTGCCCTCAACG ACCACTTT	CACCCTGTTGCT GTAGCCAAA
GPX4	glutathione peroxidase 4	NM_001039847.2	hsa00480	CGCTGTGGAAGTG GATGAAGAT	AGCCGTTCTTGTC GATGAGGA
GPX1	glutathione peroxidase 1	NM_000581.2	hsa00480	CGATGTTGCCTG GAACTTTGAG	ATGTCAATGGT CTGGAAGCGG
KEAP1	kelch like ECH associated protein 1	NM_012289	hsa04120	CAACTTCGCTGA GCAGATTGGC	TGATGAGGGTC ACCAGTTGGCA
NRF2	nuclear factor. erythroid 2-like 2	NM_001145412.3	hsa04141	CCAAAGAGCAG TTCAATGAAGC	GCAGCCACTTT ATTCTTACCCC
SOD1	superoxide dismutase 1	NM_000454.4	hsa04146	GGATGAAGAGA GGCATGTTGGA	TAGACACATCG GCCACACCAT
CAT	catalase	NM_001752.3		TGCTGAGAAGCCTA AGAATGCG	ACAGATTTGCCTTC TCCCTTGC

3.2.2. Storage of Nanoparticles at 4°C

Table S2. Stability study of the physicochemical characteristics of the nanocarriers during storage at 4°C.

Sample	Time (days)	Mean hydrodynamic diameter (nm)	PdI	ζ – potential (mV)	CUR content (mg/mL)
**SLN**	1	131.43 ± 9.94	0.24 ± 0.03	-59.58 ± 0.55	-
	8	134.36 ± 7.31	0.25 ± 0.01	-60.37 ± 1.42	-
	15	134.72 ± 11.30	0.26 ± 0.03	-59.80 ± 1.15	-
	30	136.91 ± 17.30	0.26 ± 0.02	-60.89 ± 1.63	-
	60	138.03 ± 9.96	0.29 ± 0.03	-57.12 ± 1.81	-
	90	129.04 ± 1.92	0.27 ± 0.03	-59.57 ± 2.01	-
**NLC**	1	127.71 ± 8.19	0.23 ± 0.01	-59.36 ± 1.48	-
	8	131.16 ± 7.08	0.22 ± 0.01	-61.59 ± 2.74	-
	15	129.39 ± 4.72	0.23 ± 0.01	-60.48 ± 1.93	-
	30	130.04 ± 8.74	0.22 ± 0.02	-62.14 ± 0.68	-
	60	132.59 ± 9.06	0.26 ± 0.04	-60.84 ± 3.00	-
	90	124.30 ± 4.23	0.24 ± 0.02	-59.52 ± 1.12	-
**NE**	1	122.60 ± 8.39	0.23 ± 0.03	-60.38 ± 2.09	-
	8	126.41 ± 7.10	0.24 ± 0.01	-58.41 ± 5.21	-
	15	125.03 ± 6.05	0.23 ± 0.01	-60.87 ± 1.23	-
	30	123.39 ± 8.59	0.24 ± 0.01	-63.12 ± 1.45	-
	60	128.28 ± 9.75	0.25 ± 0.03	-60.51 ± 2.51	-
	90	118.58 ± 2.28	0.24 ± 0.01	-61.47 ± 4.23	-
**SLN.CUR**	1	134.32 ± 7.66	0.26 ± 0.00	-59.79 ± 3.35	0.437 ± 0.001
	8	135.44 ± 7.78	0.29 ± 0.02	-59.93 ± 1.87	0.429 ± 0.005
	15	127.57 ± 10.05	0.26 ± 0.01	-58.74 ± 1.19	0.425 ± 0.006
	30	132.27 ± 11.57	0.26 ± 0.01	-59.20 ± 2.71	0.424 ± 0.002
	60	136.69 ± 13.54	0.31 ± 0.06	-59.24 ± 0.82	0.412 ± 0.010
	90	127.80 ± 4.61	0.29 ± 0.06	-55.12 ± 3.99	0.407 ± 0.005
**NLC.CUR**	1	134.23 ± 5.32	0.23 ± 0.02	-62.22 ± 1.82	0.468 ± 0.007
	8	132.26 ± 5.53	0.23 ± 0.01	-62.46 ± 2.86	0.470 ± 0.001
	15	129.62 ± 7.94	0.24 ± 0.01	-59.90 ± 0.25	0.468 ± 0.004
	30	132.08 ± 10.04	0.24 ± 0.01	-59.30 ± 1.50	0.466 ± 0.004
	60	135.44 ± 12.44	0.26 ± 0.02	-57.24 ± 2.12	0.465 ± 0.006
	90	125.87 ± 3.52	0.24 ± 0.01	-59.61 ± 0.48	0.461 ± 0.009
**NE.CUR**	1	128.23 ± 1.97	0.24 ± 0.02	-61.70 ± 2.79	0.447 ± 0.005
	8	127.73 ± 4.55	0.25 ± 0.01	-59.11 ± 2.87	0.443 ± 0.006
	15	124.10 ± 2.11	0.24 ± 0.01	-61.80 ± 1.19	0.440 ± 0.002
	30	127.16 ± 6.22	0.24 ± 0.01	-62.23 ± 1.87	0.434 ± 0.006
	60	130.22 ± 12.36	0.26 ± 0.02	-60.47 ± 1.77	0.411 ± 0.006
	90	131.77 ± 3.87	0.29 ± 0.02	-62.36 ± 1.57	0.394 ± 0.005

3.2.3. Accelerated Aging Test on Nanoparticles

Table S3. Changes in Physicochemical Characteristics of Nanocarriers During the Accelerated Aging Test.

Sample	Time (days)	Mean hydrodynamic diameter (nm)	PdI	ζ – potential (mV)	CUR content (mg/mL)
**SLN**	1	131.43 ± 9.94	0.24 ± 0.03	-59.58 ± 0.55	-
	7	121.28 ± 6,53	0.23 ± 0.01	-59.53 ± 0.58	-
**NLC**	1	127.71 ± 8.19	0.23 ± 0.01	-59.36 ± 1.48	-
	7	114.89 ± 7.29	0.23 ± 0.02	-59.81 ± 2.58	-
**NE**	1	122.60 ± 8.39	0.23 ± 0.03	-60.38 ± 2.09	-
	7	114.20 ± 7.25	0.23 ± 0.01	-61.71 ± 0.52	-
**SLN.CUR**	1	134.32 ± 7.66	0.26 ± 0.00	-59.79 ± 3.35	0.437 ± 0.001
	7	118.89 ± 13.03	0.24 ± 0.02	-57.08 ± 0.35	0.415 ± 0.009
**NLC.CUR**	1	134.23 ± 5.32	0.23 ± 0.02	-62.22 ± 1.82	0.468 ± 0.007
	7	115.88 ± 13.13	0.23 ± 0.01	-60.08 ± 0.88	0.442 ± 0.014
	7	109.10 ± 10.64	0.23 ± 0.03	-61.33 ± 0.75	0.424 ± 0.006

3.3. Evaluation of Antioxidant Activity of Nanoparticles

3.3.1 Antioxidant Activity via DPPH Radical Scavenging Mechanism

Table S4. Concentrations of Aqueous (A) and Methanolic (B) Ascorbic Acid Solutions and Corresponding DPPH Radical Inhibition Percentage.

Solution	Concentration (μg/mL)	% RSA
A	70	94.48
	50	56.66
	20	23.99
	10	9.67
	5	2.44
	2.5	0.84
	1.25	0.04
B	70	93.81
	50	72.86
	20	26.68
	10	14.16
	5	6.65
	2.5	1.58

Table S5. Antioxidant Activity of CUR-Loaded Nanocarriers (SLN, NLC, NE) and Free CUR expressed as DPPH Radical Inhibition Percentage (% RSA) and Ascorbic Acid Equivalents (μg/mL). Initial Cur concentration in all samples: 50 μg/mL.

Sample	% RSA	Ascorbic Acid Equivalents (μg/mL)
SLN.CUR	17.75 ± 0.94	15.83 ± 0.71
NLC.CUR	16.23 ± 1.88	14.69 ± 1.41
NE.CUR	14.03 ± 3.92	13.04 ± 2.94
CUR	39.40 ± 1.01	28.74 ± 0.73
Ascorbic Acid aqueous solution	56.65 ± 4.41	-
Ascorbic Acid methanolic solution	72.85 ± 2.16	-

3.3.2 Antioxidant Activity via Iron Cation Reduction Mechanism (FRAP Method)

Table S6. Concentrations of Aqueous (A) and Methanolic (B) Ascorbic Acid Solutions and Corresponding Absorbance at 595 nm for Standard Curve.

Solution	Concentration (μg/mL)	Absorbance
A	100	0.429
	70	0.244
	50	0.123
	20	0.029
B	100	0.274
	70	0.230
	50	0.171
	20	0.088
	10	0.054
	5	0.036

3.5. Effect of Nanoparticles on SC Permeability

Table S7. Antioxidant Activity of Nanocarriers SLN.CUR, NLC.CUR, NE.CUR, and Free CUR Expressed as Ascorbic Acid Equivalents (μg/mL). Initial Cur concentration in all samples: 50 μg/mL.

Sample	Concentration (μg/mL)	Ascorbic Acid Equivalents (μg/mL)
SLN.CUR	50	45.52 ± 1.20
NLC.CUR	50	44.93 ± 0.30
NE.CUR	50	42.44 ± 1.77
CUR	50	12.13 ± 2.47

Table S8. Quantitative Determination of CUR in Nanocarriers After Tape Stripping from the Skin of Healthy Volunteers.

Sample	Time (min)	Tape number	CUR Amount (µg/cm²)	Total CUR Amount (µg/cm²)	CUR Detected Percentage (%)
SLN.CUR	30	1	0.11 ± 0.03	0.31 ± 0.04	37.29 ± 13.18
		2+3	0.10 ± 0.03		31.38 ± 6.53
		4+5	0.10 ± 0.05		31.33 ± 14.54
	60	1	0.11 ± 0.02	0.30 ± 0.04	36.42 ± 10.60
		2+3	0.09 ± 0.02		30.27 ± 5.89
		4+5	0.10 ± 0.04		33.31 ± 10.45
	120	1	0.08 ± 0.03	0.27 ± 0.04	31.41 ± 11.39
		2+3	0.10 ± 0.02		36.05 ± 8.85
		4+5	0.09 ± 0.03		32.54 ± 8.49
NLC.CUR	30	1	0.12 ± 0.04	0.33 ± 0.06	35.18 ± 8.58
		2+3	0.12 ± 0.04		36.75 ± 8.38
		4+5	0.09 ± 0.03		28.08 ± 11.16
	60	1	0.10 ± 0.03	0.29 ± 0.07	34.03 ± 4.98
		2+3	0.10 ± 0.04		33.45 ± 7.22
		4+5	0.09 ± 0.03		32.52 ± 8.53
	120	1	0.08 ± 0.02	0.24 ± 0.06	32.13 ± 3.80
		2+3	0.09 ± 0.02		37.04 ± 10.36
		4+5	0.08 ± 0.03		30.83 ± 10.27
NE.CUR	30	1	0.12 ± 0.04	0.32 ± 0.13	39.08 ± 3.30
		2+3	0.11 ± 0.03		34.51 ± 5.79
		4+5	0.09 ± 0.06		26.40 ± 7.45
	60	1	0.10 ± 0.04	0.29 ± 0.10	34.00 ± 6.47
		2+3	0.11 ± 0.03		40.14 ± 11.48
		4+5	0.08 ± 0.04		25.86 ± 6.08
	120	1	0.09 ± 0.04	0.27 ± 0.10	30.22 ± 5.51
		2+3	0.10 ± 0.03		39.49 ± 9.67
		4+5	0.09 ± 0.04		30.28 ± 5.53

3.1.2. Morphological Study of Nanoparticles by Transmission Electron Microscopy

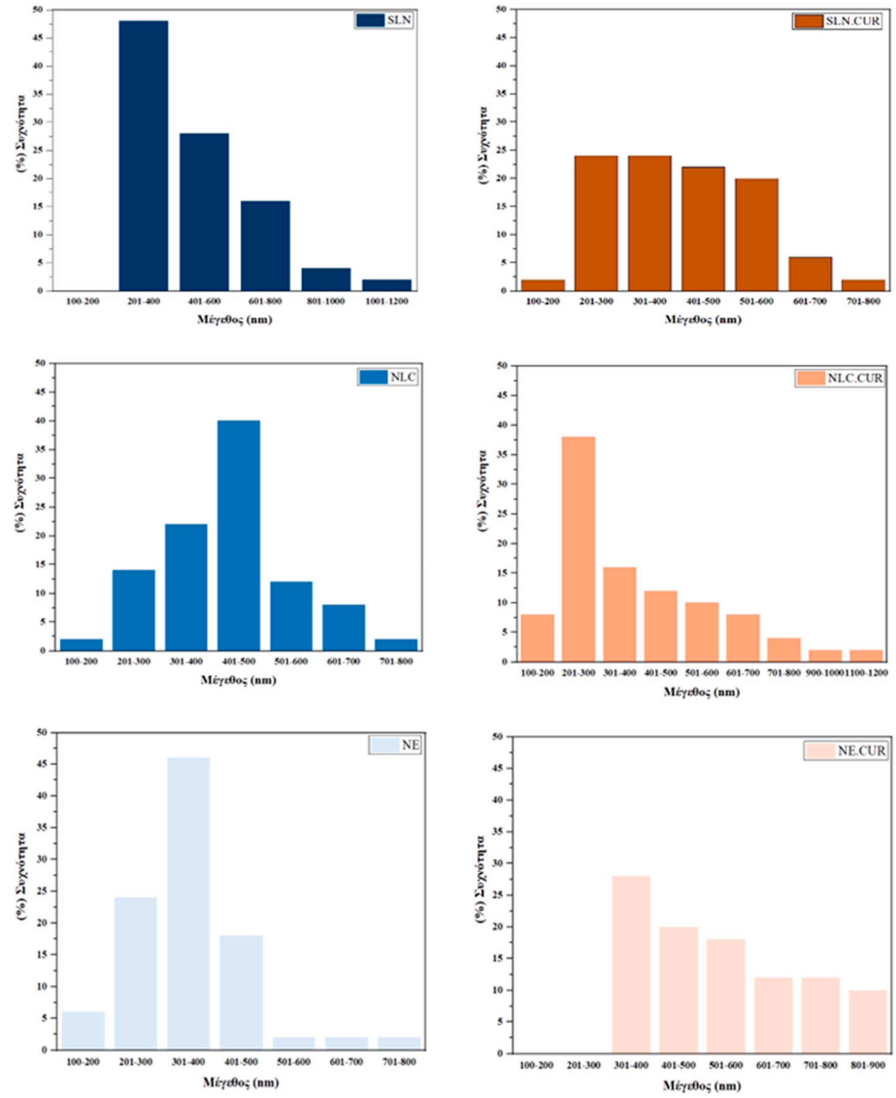

Figure S1. Size distribution for empty nanoparticles (left):and loaded with CUR (right), as derived from the analysis of TEM microscope images.

3.4.1. In vitro cell viability assay

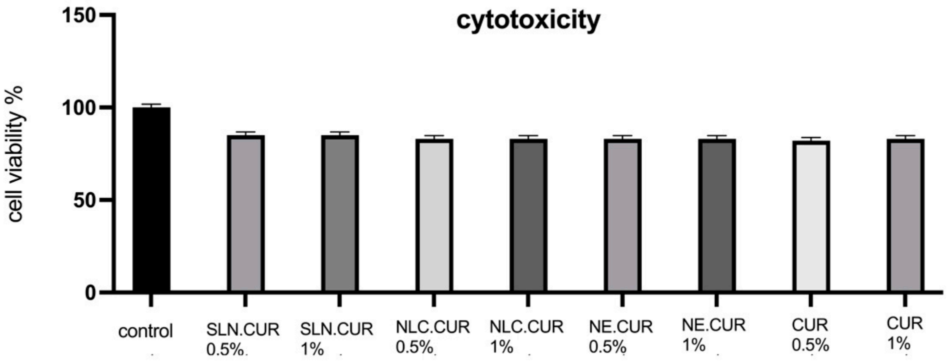

Figure S2. Cell viability assessments for control, SLN (SLN.CUR), NLC (NLC.CUR), NE (NE.CUR) and curcumin (CUR) in different concentrations (0.5%,1%). The results are presented as a percentage ± SD respect to control and represent the mean ± SEM of three independent experiments
